# Carbon Ion Therapy for Early-Stage Non-Small-Cell Lung Cancer

**DOI:** 10.1155/2014/727962

**Published:** 2014-09-11

**Authors:** Yusuke Demizu, Osamu Fujii, Hiromitsu Iwata, Nobukazu Fuwa

**Affiliations:** ^1^Department of Radiology, Hyogo Ion Beam Medical Center, 1-2-1 Kouto, Shingu-cho, Tatsuno, Hyogo 679-5165, Japan; ^2^Department of Radiation Oncology, Nagoya Proton Therapy Center, Nagoya City West Medical Center, 1-1-1 Hiratecho, Kita-ku, Nagoya, Aichi 462-8508, Japan

## Abstract

Carbon ion therapy is a type of radiotherapies that can deliver high-dose radiation to a tumor while minimizing the dose delivered to the organs at risk; this profile differs from that of photon radiotherapy. Moreover, carbon ions are classified as high-linear energy transfer radiation and are expected to be effective for even photon-resistant tumors. Recently, high-precision radiotherapy modalities such as stereotactic body radiotherapy (SBRT), proton therapy, and carbon ion therapy have been used for patients with early-stage non-small-cell lung cancer, and the results are promising, as, for carbon ion therapy, local control and overall survival rates at 5 years are 80–90% and 40–50%, respectively. Carbon ion therapy may be theoretically superior to SBRT and proton therapy, but the literature that is currently available does not show a statistically significant difference among these treatments. Carbon ion therapy demonstrates a better dose distribution than both SBRT and proton therapy in most cases of early-stage lung cancer. Therefore, carbon ion therapy may be safer for treating patients with adverse conditions such as large tumors, central tumors, and poor pulmonary function. Furthermore, carbon ion therapy may also be suitable for dose escalation and hypofractionation.

## 1. Introduction

Carbon ion therapy, also known as carbon ion radiation therapy, is a type of radiotherapies that is categorized as particle therapy. While photons are used for conventional radiotherapy, beams with completely different characteristics (such as protons and carbon ions) are used in particle therapy. Heavy ion radiotherapy is a synonym of carbon ion therapy in current clinical practice.

At present, approximately 40 particle therapy centers are available worldwide. Only 8 have carbon ion therapy facilities (4 in Japan, 2 in Germany, 1 in China, and 1 in Italy), and the remainder have proton therapy facilities (current information available at the website of the Particle Therapy Co-Operative Group: http://www.ptcog.ch/). This disparity is likely to exist because proton therapy facilities are smaller and have lower installation costs and operating costs. For example, installation costs approximately 70 million USD for proton facilities are compared with approximately 140 million USD for carbon ion facilities. Furthermore, rotating gantries are basically only available for proton therapy facilities; Heidelberg Ion Beam Therapy Center (HIT), Germany, is the only institution that possesses a rotating gantry which can be used for carbon ion therapy.

## 2. History of Carbon Ion Therapy

The history of particle therapy began with proton therapy at Lawrence Berkeley Laboratory, 1954 [1]. After the trials with several types of particle therapy, including neutron, pion, helium ion, and neon ion, carbon ion therapy started at the National Institute of Radiological Sciences (NIRS), Japan, 1994 [2]. Among the various types of ion species, carbon ions were chosen for therapy because the biologically expressed dose distribution is assumed to be superior to other types of ion species. Additionally, the amount of high-linear energy transfer (LET) components is assumed to be sufficient to ensure a benefit by controlling radioresistant tumors. The details of physical and biological characteristics of carbon ion therapy are described below. Excellent clinical outcomes from NIRS led to the subsequent carbon ion therapy facilities, such as Gesellschaft für Schwerionenforschung, Germany, 1997; Hyogo Ion Beam Medical Center (HIBMC), Japan, 2002; Institute of Modern Physics, China, 2006; HIT, Germany, 2009; Gunma University Heavy Ion Medical Center, Japan, 2010; Centro Nazionale di Adroterapia Oncologica (CNAO), Italy, 2012; and Saga Heavy Ion Medical Accelerator, Tosu, Japan, 2013. Among these carbon ion therapy centers, HIBMC, HIT, and CNAO also have proton therapy facilities. More than 13,000 patients have been treated with carbon ion therapy around the world as of the end of 2013. Several carbon ion therapy facilities are under construction or in the planning phase worldwide, primarily in Japan.

## 3. Physical Characteristics of Carbon Ion Therapy

**Figure 1:**
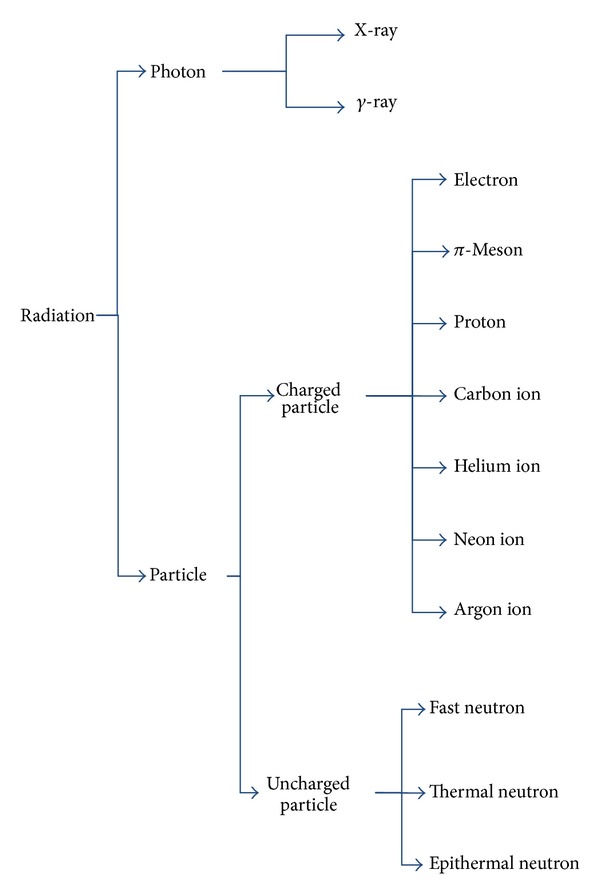
Types of radiation.

**Figure 2:**
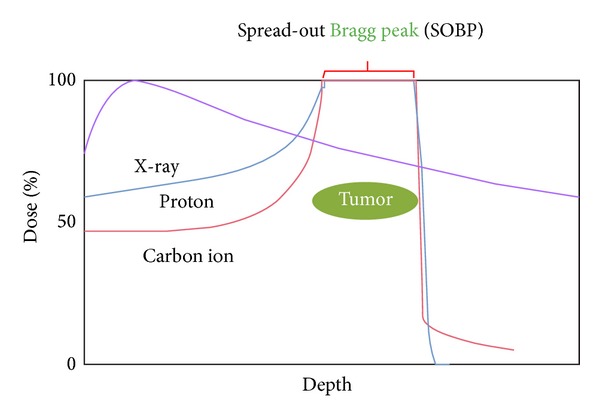
Dose distributions of X-rays, protons, and carbon ions.

Photons consist of waves of light and do not possess an electric charge or mass, whereas charged particles such as protons and carbon ions possess electric charge and mass (Figure 1). Photons emit maximal energy near the body surface; this energy decreases gradually and passes through the entire thickness of body structures. In contrast, charged particles emit a relatively low dose near the body surface and deposit their maximum energy just before stopping in the deep interior of the body, an effect known as the Bragg peak. By modifying this peak according to the position and size of the tumor into a spread-out Bragg peak (SOBP) [3], it is possible to deliver high-dose radiation to a tumor while minimizing the dose delivered to the organs at risk (Figure 2).

**Figure 3:**
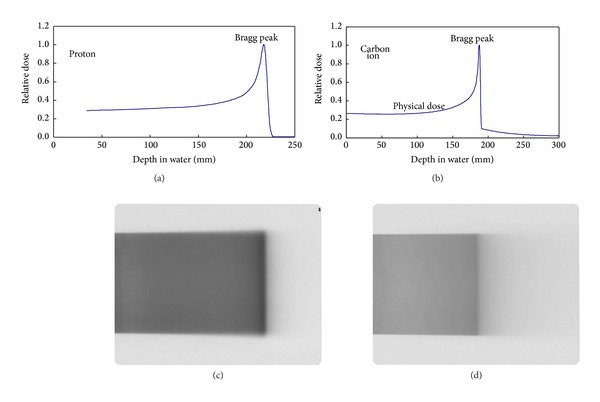
Differences in the dose distributions of proton ((a), (c)) and carbon ion ((b), (d)) monoenergetic beams ((a), (b) calculated and measured depth-dose curves; ((c), (d)) film densitometry).

Although both proton therapy and carbon ion therapy are charged particle therapies, there are slight differences in their physical characteristics. With respect to monoenergetic beams, carbon ion therapy shows a superior penumbra compared with proton therapy and low-dose leakage (<10%) on the distal side of the Bragg peak, unlike proton therapy (due to nuclear spallation reactions) (Figure 3). However, the latter issue does not impact practice because two or more portals are typically used in a clinical setting. The largest difference in the mechanical aspects of these approaches is the availability of a rotating gantry, which can rotate 360 degrees and allows the tumor to be irradiated from arbitrary angles.  Table 1 shows a comparison between protons and carbon ions at HIBMC.

## 4. Biological Characteristics of Carbon Ion Therapy

Carbon ions, which are classified as high LET radiation, show a high ionization density and a high rate of DNA damage caused by the direct action of radiation. Carbon ions are likely to induce DNA double-strand breaks, which are difficult to repair and frequently lead to cell death [4]. Thus, carbon ions have the following biological characteristics and are expected to be effective even for photon-resistant tumors. First, they have a high relative biological effectiveness (RBE), showing 1.2- to 3.5-fold greater biological effects compared with equal physical doses of photons, depending on the position of the SOBP. Second, they have a low oxygen enhancement ratio (OER), meaning that they are effective for treating photon-resistant hypoxic cells. Third, they are less dependent on the cell cycle, suggesting that they may be effective for treating photon-resistant late-S phase cells. The modes of carbon-ion-induced cell death and inactivation include apoptosis, necrosis, autophagy, premature senescence, accelerated differentiation, delayed reproductive death of progeny cells, and bystander cell death [4].

In addition to the excellent local effects, carbon ion therapy may suppress the metastatic potential of cancer cells. Based on* in vitro* and* in vivo* experiments, Ogata et al. suggested that carbon ion irradiation suppresses metastatic potential even at low doses, whereas photon irradiation promotes cell migration and invasive capabilities at a lower dose level [5]. They also provided preclinical evidence that carbon ion therapy is potentially superior to conventional photon therapy in preventing effects on metastases of irradiated malignant tumor cells. An* in vitro* study conducted by Akino et al. investigated the effects of carbon ion irradiation on the metastatic capacity in association with gene expression of non-small-cell lung cancer (NSCLC) cells [6]. The results showed that carbon ion irradiation effectively suppressed the metastatic potential of NSCLC cells. Carbon ion irradiation also had different effects on gene expression, and the downregulation of a gene that is overexpressed in the majority of primary NSCLC was induced by carbon ion irradiation.

Notably, protons are classified as low LET radiation, and their biological effects are considered to be nearly the same as those of photons (RBE = 1.1) [7].

## 5. Carbon Ion Therapy for Early-Stage Non-Small-Cell Lung Cancer

Surgical resection with lobectomy has been the standard treatment of choice for early-stage NSCLC: overall survival (OS) rates at 5 years for stages I and II disease are 70% and 40–50%, respectively [8–10]. However, radiotherapy is an option for patients who are not suitable for surgery or refuse it. Recently, stereotactic body radiotherapy (SBRT) using photons has been increasingly used for such patients [11–14]. Another type of high-precision radiotherapy for early-stage NSCLC is particle therapy, including proton therapy [15–20] and carbon ion therapy [17, 19–25]. In this special issue, we focus on carbon ion therapy, and additional reports describe the details of SBRT and proton therapy.

Studies analyzing carbon ion therapy for early-stage NSCLC are summarized in Table 2. Only two Japanese institutions have published these data sets: NIRS [21–25] and HIBMC [17, 19, 20]. In terms of treatment system, NIRS uses horizontal and vertical fixed portals with semicylindrically shaped rotary capsule set on a treatment couch to reduce the disadvantage of unavailability of rotating gantry, whereas HIBMC uses horizontal, vertical, and 45-degree oblique fixed portals. Respiratory-gated irradiation systems are employed at both institutions to minimize respiratory movements of the tumor and reduce treatment volume.

The NIRS group has published 5 reports. Miyamoto et al. started a phase I/II trial of carbon ion therapy for stage I NSCLC using 18-fraction regimens based on their years of experience with fast neutron therapy, which is also high LET radiation [21]. They conducted a dose escalation study from 59.4 to 95.4 Gy (RBE). (The particle beam dose is reported in Gy (RBE), which is defined as the physical dose multiplied by the RBE of the protons or carbon ions.) Then, they moved to 9-fraction regimens, with dose escalation from 68.4 to 79.2 Gy (RBE). The 5-year local control (LC) rates of the 18- and 9-fraction regimens were 64% and 84%, respectively (76% for all patients). The hypofractionated regimens showed much better LC. Grade 2 radiation pneumonitis occurred at a rate of 2/3 at the 79.2 Gy (RBE) dose level in 9-fraction regimens; therefore they concluded that 72 Gy (RBE), a dose 10% below 79.2 Gy (RBE), in 9 fractions was recommended regimen for a phase II study. The phase II study treated 50 patients with 51 lesions and showed an excellent 5-year LC rate of 94.7% without grade 3 or greater radiation pneumonitis [22]. The 5-year OS and cause-specific survival (CSS) rates were 50.0% (IA 55.2%; IB 42.9%) and 75.7% (IA 89.4%; IB 55.1%), respectively. Patients with stage IA disease showed significantly better OS and CSS compared to those with stage IB. Next, they conducted an additional phase II study using a regimen of 4 fractions during 1 week [23]. Seventy-nine patients with 80 lesions were treated with a fixed dose of 52.8 Gy (RBE) for stage IA and 60 Gy (RBE) for stage IB. The 5-year LC and OS rates were 90% (T1 98%; T2 80%) and 45% (IA 62%; IB 25%), respectively. No grade 3 or greater toxicities were detected. Although the patients treated in this study were approximately 10 years older than the patients treated by surgery, carbon ion therapy achieved impressive results. Therefore, Sugane et al. next focused on 28 patients aged 80 years and older (median 82 years, range 80–86 years) with stage I NSCLC who underwent carbon ion therapy with 52.8–72 Gy (RBE) in 4–9 fractions [24]. Outcomes were focused on the effectiveness of carbon ion therapy in treating their lung cancer and the impact on their activity of daily life (ADL). Pulmonary function was determined to be too poor for tumor resection by the referring surgeons in 16 patients, and 7 patients refused due to advanced age and poor systemic conditions. Five patients suffered from other diseases, including cardiovascular disease. The 5-year LC and OS rates were 95.8% and 30.7%, respectively. No grade 3 or greater toxicities occurred and no patients started home oxygen therapy or had decreased ADL. In their latest report, Takahashi et al. showed the preliminary results of a phase I/II trial as a dose escalation study using a single fraction [25]. The initial total dose was 28 Gy (RBE) and escalated in increments of 2 Gy (RBE), up to 50 Gy (RBE). For 151 patients treated with 36–50 Gy (RBE), the 5-year LC and OS rates were 79.2% and 55.1%, respectively. No grade 3 or greater toxicities were observed.

The HIBMC group has published 3 reports. HIBMC was established as the first institution in the world that could use both carbon ion therapy and proton therapy, 2001, and more than 6,100 patients have been treated as of the end of 2013. Thus, our studies include the results of both carbon ion therapy and proton therapy; however, here we describe only carbon ion therapy findings. At HIBMC, the policy for selecting beam type was based partly on the availability of the particle beams (between April 2003 and March 2005, only proton therapy was available). In April 2005, carbon ion therapy became available; thereafter, treatment plans for both proton therapy and carbon ion therapy were made for every patient. Then, the dose-volume histograms were compared, and the more suitable modality (proton therapy or carbon ion therapy) was determined and used for each patient. Iwata et al. reported the clinical outcome of carbon ion therapy for 23 patients with stage I NSCLC [17]. The protocol of 52.8 Gy (RBE) in 4 fractions was employed according to the NIRS study [23]. The 3-year LC and OS rates were 86% and 86%, respectively. No grade 3 or greater toxicities were observed. In the second report by Iwata et al. [19], their hypothesis was that particle therapy might be superior to SBRT in T2 (>3 cm) patients because it is rather difficult to treat T2 tumors with SBRT. Twenty-seven patients with T2 tumors were treated with 52.8–68.4 Gy (RBE) in 4–10 fractions. The 4-year LC and OS rates were 75% and 55%, respectively. Severe radiation pneumonitis (grade 3) was noted in 2 patients (7%). Both had T2b (>5 cm) disease and idiopathic pulmonary fibrosis with very poor respiratory function. They concluded that particle therapy was well tolerated and effective for T2N0 M0 NSCLC. The most recent report by Fujii et al. included 41 patients treated with 52.8–70.2 Gy (RBE) in 4–26 fractions [20]. The 3-year LC and OS rates were 78% and 76%, respectively. Severe radiation pneumonitis (grade 3) was observed in 2 patients (5%). In this study, they retrospectively compared the clinical outcomes of carbon ion therapy with those of proton therapy for stage I NSCLC and found no significant difference between the two groups.

Overall, the results of carbon ion therapy for early-stage NSCLC are promising and similar to those of SBRT or proton therapy in terms of LC, OS, and late toxicity. This result is not entirely expected because carbon ions are high LET radiation and could be expected to yield better outcomes. Grutters et al. reported a meta-analysis that compared the effectiveness of radiotherapy with photons, protons, and carbon ions for stage I NSCLC [26]. They concluded the following. (1) The corrected pooled 2- and 5-year OS estimates were 53% and 19%, respectively, for conventional radiotherapy; 70% and 42%, respectively, for SBRT; 61% and 40%, respectively, for proton therapy; and 74% and 42%, respectively, for carbon ion therapy. (2) The OS for patients treated with conventional radiotherapy was significantly shorter than that of patients receiving SBRT, proton therapy, or carbon ion therapy at both 2 and 5 years. (3) SBRT, proton therapy, and carbon ion therapy did not have significantly different 2- or 5-year OS rates. (4) The occurrence of severe adverse events (grades 3–5) was infrequent for all treatment modalities. From the literature currently available, it is difficult to claim that carbon ion therapy provides clinical outcomes that are superior to those of other high-precision radiotherapies such as SBRT and proton therapy.

**Figure 4:**
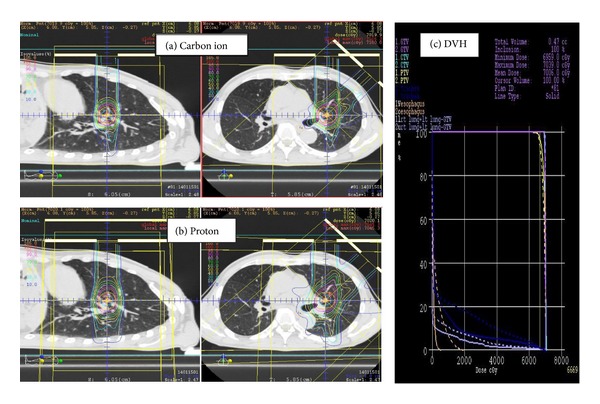
Comparison of the carbon ion (a) and proton (b) treatment plans for central-type T1aN0M0 non-small-cell lung cancer. The solid and dashed curves represent the carbon ion treatment plan and proton treatment plan, respectively, in the dose-volume histogram (DVH) (c). The carbon ion was selected for this patient.

Therefore, it is reasonable to examine the potential advantages of carbon ion therapy. It is unquestionable that carbon ion therapy shows a better dose distribution than SBRT in terms of the low-dose irradiated volume of the lung [27], but less is known about comparing carbon ion therapy with proton therapy. From our experience at HIBMC, where we routinely make both carbon ion and proton therapy plans for each patient, carbon ion therapy demonstrates a better dose distribution in most patients with early-stage lung cancer. A representative comparison of carbon ion therapy and proton therapy plans for central-type T1aN0M0 NSCLC is shown in Figure 4. In this case, it is possible for carbon ion therapy to reduce the doses to the lung, left main bronchus, and esophagus while achieving an equal coverage of target volumes as proton therapy. This superiority of carbon ion therapy to SBRT and proton therapy in dose distribution leads to several possible benefits. First, carbon ion therapy could be safer for treating patients with adverse conditions such as large tumors (e.g., T2), central tumors, or poor lung function. When treating large or central tumors, relatively large volumes of the lung, main bronchus, trachea, and esophagus, for example, are irradiated, and it is therefore preferable to avoid unnecessary irradiation as much as possible. When treating patients with poor lung function due to chronic obstructive pulmonary disease or interstitial pneumonitis, it is crucial to keep the lung dose as low as possible. Second, carbon ion therapy may be suitable for dose escalation and hypofractionation. Dose escalation would be warranted to improve local control, and the superb dose distribution of carbon ion therapy is advantageous in terms of safety. Hypofractionation is beneficial for both patients and health professionals because of the shortening of overall treatment time. However, from the perspective of radiation biology, a larger fraction size leads to an increase in late toxicities, and a smaller fraction number weakens the merits of fractionated irradiation by allowing the reoxygenation and redistribution of the cell cycle. Basic research studies have shown that carbon ion therapy shows low OER and low dependency on the cell cycle (see the chapter of biological characteristics of carbon ion therapy); therefore, the above biological disadvantages would not be the case. In fact, the NIRS group has successfully reduced the number of fractions and reached an ultimate single-fraction regimen, up to a total dose of 50 Gy (RBE) [21–23, 25]. Conversely, SBRT using 54 Gy in 3 fractions revealed a relatively high rate (16.4%) of ≥grade 3 late toxicities [13].

**Figure 5:**
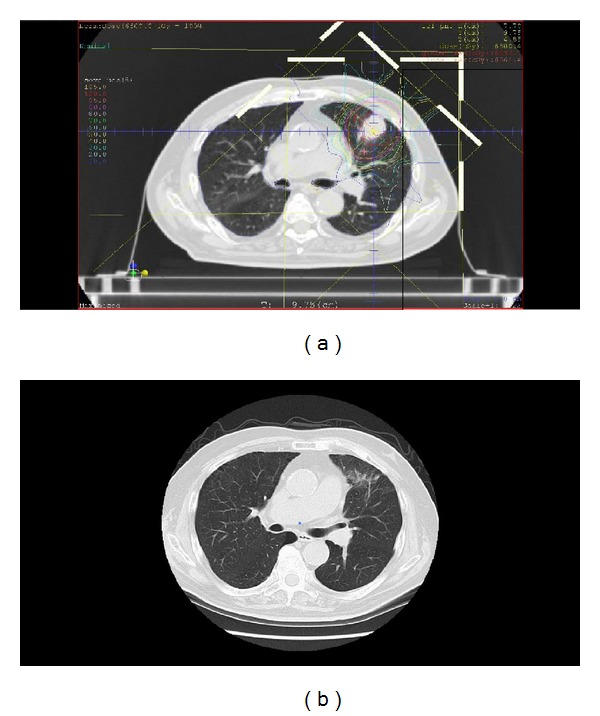
A patient with peripheral-type T2aN0M0 non-small-cell lung cancer that was treated with 66 Gy (RBE) of carbon ion therapy in 10 fractions. (a) Dose distribution. (b) A computed tomography image 5 months after carbon ion therapy.

Figure 5 demonstrates a case of an 83-year-old male with peripheral-type T2aN0M0 NSCLC. Surgical resection and chemotherapy were contraindicated for this patient because of advanced age, poor lung function, chronic renal failure, and diabetes mellitus. He was treated with 66 Gy (RBE) of carbon ion therapy in 10 fractions (Figure 5(a)). The patient's acute reaction consisted only of grade 1 dermatitis. Five months later, the tumor showed a complete response, and grade 1 radiation pneumonitis was observed (Figure 5(b)). He is alive without recurrence 9 months after carbon ion therapy.

## 6. Future Perspective

To further improve treatment outcomes, new irradiation technologies such as layer-stacking [28] and scanning [29, 30] are emerging. Although conventional passive beam irradiation benefits from relatively simple treatment planning requirements, one disadvantage of conventional beam irradiation is the significantly excessive dose delivered to the normal tissues along the entrance to the target. Layer-stacking and scanning to a greater extent can reduce this excessive dose, but it is challenging to adopt these technologies to moving targets such as lung tumors. If the day comes when these technologies are available in the clinical settings, carbon ion therapy will be the more effective and safer treatment option for early-stage NSCLC.

The progress of other treatment modalities such as SBRT, proton therapy, radiofrequency ablation, cryoablation, and less invasive surgery for early-stage NSCLC is also likely. It will be crucial to choose an appropriate modality for each case with careful consideration.

## 7. Conclusions

Carbon ion therapy for early-stage NSCLC has shown promising results and may be theoretically superior to other high-precision radiotherapy approaches such as SBRT and proton therapy in both physical and biological aspects. However, the currently available literature does not show a statistically significant clinical difference among these treatment options. Carbon ion therapy demonstrates a better dose distribution than SBRT (and even proton therapy) in most cases of early-stage lung cancer; thus, carbon ion therapy may be safer for treating patients with adverse conditions such as large tumors (e.g., T2), central tumors, and poor pulmonary function. Carbon ion therapy may also be suitable for dose escalation and hypofractionation. Prospective randomized controlled trials are warranted to elucidate whether there is truly no difference in clinical outcomes among SBRT, proton therapy, and carbon ion therapy.

## Figures and Tables

**Table 1:** Comparison of the physical aspects of protons and carbon ions.

	Protons	Carbon ions
Rotating gantry	Available	Not available (fixed portals only)
Penumbra	Inferior	Superior
Range	Longer	Shorter

**Table 2:** Studies of carbon ion therapy for early-stage non-small-cell lung cancer.

Author	Institute	Year	Number of patients	Age (years)	Number of lesions	T1	T2	Total dose [Gy (RBE)]	Number of fractions	Median FU (months)	Local control	Overall survival	Toxicity (≥grade 3)
Miyamoto et al. [21]	NIRS	2003	81	Mean 72	82	41	41	59.4–95.4	9–18	52.6	76% (5-yr)	42% (5-yr)	Lung 3.7%
Miyamoto et al. [22]	NIRS	2007	50	Mean 74.1	51	30	21	72	9	59.2	94.7% (5-yr)	50.0% (5-yr)	Skin 2%
Miyamoto et al. [23]	NIRS	2007	79	Mean 74.8	80	42	37	52.8–60	4	38.6	90% (5-yr)	45% (5-yr)	0%
Sugane et al. [24]	NIRS	2009	28	Mean 82∗	29	12	17	52.8–72	4–9	NA	95.8% (5-yr)	30.7% (5-yr)	0%
Takahashi et al. [25]	NIRS	2014	151	Mean 73.9	151	91	60	36–50	1	45.6	79.2% (5-yr)	55.1% (5-yr)	0%
Iwata et al. [17]	HIBMC	2010	23	Median 75	23	15	8	52.8	4	30.5^†^	86% (3-yr)	86% (3-yr)	0%
Iwata et al. [19]	HIBMC	2013	27	Median 75^‡^	27	0	27	52.8–68.4	4–10	44^†^	75% (4-yr)^§^	55% (4-yr)^§^	Lung 7%, skin 7%^||^
Fujii et al. [20]	HIBMC	2013	41	Median 76	41	26	15	52.8–70.2	4–26	39	78% (3-yr)	76% (3-yr)	Lung 5%, skin 4%

Gy: gray; RBE: relative biological effectiveness; FU: follow-up; NIRS: the National Institute of Radiological Sciences; yr: year; NA: not available; HIBMC: Hyogo Ion Beam Medical Center.

∗80 years and older only.

^†^The median follow-up periods for all patients including both proton and carbon ion groups.

^‡^The median age for all patients including both proton and carbon ion groups.

^§^Values determined by reading graphs.

^||^The rate for all patients including both proton and carbon ion groups.
